# Targeting the Opioid System in Cardiovascular Disease: Liver Proteomic and Lipid Profile Effects of Naloxone in Atherosclerosis

**DOI:** 10.3390/biomedicines13081802

**Published:** 2025-07-23

**Authors:** Kinga Jaskuła, Agata Nawrocka, Piotr Poznański, Aneta Stachowicz, Marzena Łazarczyk, Mariusz Sacharczuk, Zbigniew Gaciong, Dominik S. Skiba

**Affiliations:** 1 Department of Experimental Genomics, Institute of Genetics and Animal Biotechnology, Polish Academy of Sciences, Postępu 36A, 05-552 Jastrzębiec, Poland; k.jaskula@igbzpan.pl (K.J.); a.nawrocka@igbzpan.pl (A.N.); m.lazarczyk@igbzpan.pl (M.Ł.); d.skiba@igbzpan.pl (D.S.S.); 2 Department of Biotechnology and Nutrigenomics, Institute of Genetics and Animal Biotechnology, Polish Academy of Sciences, Postępu 36A, 05-552 Jastrzębiec, Poland; p.poznanski@igbzpan.pl; 3 Department of Pharmacology, Faculty of Medicine, Jagiellonian University Medical College, 31-008 Krakow, Poland; aneta.stachowicz@uj.edu.pl; 4 Department of Pharmacodynamics, Faculty of Pharmacy, Warsaw Medical University, Banacha 1, 02-697 Warsaw, Poland; 5 Department and Clinic of Internal Diseases, Hypertension and Angiology, Medical University of Warsaw, Banacha 1A Street, 02-097 Warsaw, Poland; zbigniew.gaciong@wum.edu.pl

**Keywords:** opioid receptors, cholesterol, atherosclerotic plaque, liver steatosis

## Abstract

**Background:** The endogenous opioid system plays a pivotal role in numerous physiological processes and is implicated in a range of diseases, including atherosclerosis, a condition contributing to nearly 50% of deaths in Western societies. **Objectives:** This study investigates the effects of opioid receptor blockade, using naloxone, on the plasma lipid profile and atherosclerosis progression. **Methods:** ApoE^−/−^ mice with advanced atherosclerosis were treated with naloxone for seven days, and the effects on atherosclerotic plaque development and liver steatosis were evaluated. **Results:** A proteomic analysis of liver samples post-treatment identified 38 proteins with altered abundance. The results revealed that naloxone treatment led to an increase in HDL cholesterol, a lipid fraction associated with protective cardiovascular effects. Furthermore, naloxone did not influence the progression of atherosclerotic plaques or the development of liver steatosis. **Conclusions:** In conclusion, while short-term naloxone treatment in mice with advanced atherosclerosis does not alter overall atherosclerotic plaque progression or liver steatosis, the observed elevation in HDL cholesterol and the extensive changes in liver protein abundance underscore the complex and multifaceted role of the opioid system in lipid metabolism and cardiovascular health. These findings provide a foundation for further exploration of opioid receptor antagonists as modulators of lipid profiles and potential contributors to cardiovascular therapy.

## 1. Introduction

The endogenous opioid system (EOS) consists of endogenous opioid peptides (such as endorphins, enkephalins, and dynorphins) that demonstrate high affinity to three main types of target receptors: mu (µ, MOR), delta (δ, DOR), and kappa (κ, KOR), respectively [1]. The main function of EOS is modulation of nociceptive signaling, including involvement in stress-induced analgesia [2,3]. However, for the past few decades, it has been known that this system plays a role in the proper functioning of many bodily systems, as well as in pathological processes [4,5,6]. Dysfunction in the opioid system may contribute to mood disorders such as depression and anxiety [7]; for instance, reduced levels of endogenous opioids have been observed in individuals with major depressive disorder [8]. Additionally, opioid receptors are also present in the gastrointestinal tract, where endogenous opioids regulate motility and secretion [9]. Therefore, dysregulation of this system can lead to conditions such as irritable bowel syndrome [10]. Endogenous opioids are also involved in the regulation of cardiovascular function, including heart rate and blood pressure [11]. An EOS malfunction in the cardiovascular system can contribute to the development of hypertension and may lead to heart failure [12,13].

Studies also indicate that EOS influences the progression and instability of atherosclerotic plaque [14,15]. It is worth mentioning that atherosclerosis is a disease that causes about 50% of all deaths in Western society [16]. Atherosclerosis is a complex disease influenced by many factors and systems [17,18,19]. The primary causes of its development are considered chronic inflammation and lipid metabolism disturbances [20]. Moreover, opioid receptors are expressed on and influence the function of many cell types in the cardiovascular system, including cardiomyocytes, endothelial cells, and smooth muscle cells, including excitation–contraction coupling and the regulation of vascular tone. In addition to regulating cardiac function through modulation of calcium metabolism, opioids also play an important role in vascular function [21]. Opioid receptors can induce vasodilation and maintain vascular tone, influencing blood pressure and blood flow. Opioid receptors on endothelial cells (ECs) communicate with vascular smooth muscle cells (VSMCs) through paracrine signaling and modulate angiogenesis, vascular tone, and vascular integrity [22].

The connection between the opioid system and atherosclerosis is also supported by studies, which describe that the chronic infusion of β-endorphin, a ligand for the µ opioid receptor, into ApoE^−/−^ mice significantly enhances the development of atherosclerotic lesions in the aorta, along with an increase in vascular inflammation [14]. Moreover, long-term pretreatment with the opioid receptor antagonist, naloxone (NLX), followed by lipopolysaccharide (LPS) treatment in young ApoE^−/−^ mice, can inhibit LPS-induced macrophage activation by significantly reducing the tumor necrosis factor α (TNFα) level in plasma, compared to mice treated with LPS and not receiving NLX [23]. Another crucial aspect in the development of atherosclerosis is cholesterol. Previous studies indicated that pretreatment with another opioid receptor antagonist, naltrexone, prevented stress-induced increases in cholesterol levels in cholesterol–cholic acid (CCA)-fed female rats [24], which suggested that EOS may play a role in the treatment of stress-induced hypercholesterolemia. Moreover, in patients with ischemic heart disease treated with δ opioid receptor agonist, levels of total cholesterol, triglycerides, and low-density lipoproteins (LDL) were reduced, which suggested that the role of the opioid system may be strongly receptor-specific [25]. The opioid receptor axis significantly impacts liver function, as well, and is involved in various cellular processes that affect liver cell survival, inflammation, and fibrosis [26]. One of the studies showed that blockade of opioid receptors with naltrexone reduced liver fibrosis and hepatic stellate cell (HSC) activation, along with reduced plasma ALP (Alkaline Phosphatase) and ALT (Alanine Transaminase) activity, but without significantly affecting liver inflammation in BDL rats (rats with ligated bile ducts) [27]. However, another study demonstrated significantly lower µ opioid receptor gene expression in the livers of patients with hepatitis C compared to control subjects. Administration of the µ opioid receptor agonist DAMGO resulted in a significant reduction in the necrotic area, along with a marked decrease in serum ALT. NLX inhibition of the µ opioid receptor in mice with healthy livers and mice with CCl4-induced hepatitis did not alter plasma liver enzyme levels or liver histological assessments, but it increased liver cytolysis and histological necrosis compared to control animals [28].

The examples presented above illustrate the extensive involvement of the EOS in various physiological and pathological processes, including cardiovascular physiology and pathology. In our research, we aimed to explore the therapeutic potential of modifying the activity of the EOS in mice with developed atherosclerosis, especially focused on investigating its influence on lipid metabolism and the development of atherosclerotic plaque. Secondly, considering the significant potential role of the EOS in regulating lipid levels, our research focused on examining the lipid profile and liver proteome, the site of cholesterol synthesis and metabolism, to gain a better understanding of the mechanisms influencing lipid regulation.

## 2. Materials and Methods

### 2.1. Animals

All the experiments were performed on 36-week-old, male B6.129P2-Apoe^tm1Unc^/J (strain no. 002052) mice on the C57BL/6J background (further referred to as ApoE^−/−^). The animals were housed in groups of 4–5 individuals and maintained in the animal facility of the Institute of Genetics and Animal Biotechnology of the Polish Academy of Sciences under standard environmental conditions (ambient temperature of 22 ± 2 °C and 55 ± 5% relative humidity) under a 12 h light/dark cycle (lights on at 7 a.m.). Access to tap water and food (LABOFEED H, Kcynia, Poland) was provided *ad libitum*. Study procedures were carried out in accordance with the ethical clearance (permission no. WAW2/093/2024) received from the II Local Ethics Committee for Experiments on Animals in Warsaw.

### 2.2. Drug and Experiment Design

The mice were assigned to a saline-treated (control, NaCl) or NLX-treated (experimental) group. Naloxone hydrochloride (NLX) was purchased from Sigma-Aldrich (St. Louis, MO, USA) and served as a non-selective opioid system antagonist. Individuals belonging to the NLX-treated group were administered daily with freshly prepared NLX dissolved in saline (0.9% NaCl) by intraperitoneal injections in a dose of 10 mg/kg for 7 days. The control group received the saline solution.

### 2.3. Lipid Profile Analysis

Blood was collected directly from the hearts of anesthetized mice into heparin-coated syringes. The blood samples were centrifuged at 3000× *g* at 4 °C for 10 min, then the plasma was collected for lipid profile analysis, including levels of total cholesterol, LDL, HDL, and triglycerides. The analyses were performed using a COBAS 6000 analyzer (Roche Diagnostics, Mannheim, Germany).

### 2.4. Oil Red O (ORO) Staining

Oil red O (ORO) staining was performed on aortic arches and right lobes of liver to visualize atherosclerotic lesions and fat deposits, respectively. The aortic arches were harvested after prior perfusion with phosphate-buffered saline (PBS), then fixed in 10% formalin for 24 h at 4 °C and stained according to an adjusted protocol [17]. Briefly, fixated aortic arches were opened and pinned onto black silicone cubes. After that, each specimen was rinsed in distilled water, followed by a quick wash in 60% isopropanol solution. Then, the aortic arches were stained in ORO working solution (0.625% ORO solution in isopropanol mixed with distilled water in a ratio of 1.5:1) for 30 min at room temperature. To remove excess stain, the specimens were washed with 60% isopropanol followed by three dips in distilled water.

The right lobe of the liver was collected and freshly frozen in −80 °C. Prior to sectioning, the sample was transferred for 30 min to −20 °C to avoid tissue crumbling during cutting. The tissue was sectioned into 6 µm thick sections on a cryostat (Jung CM1800, Leica, Düsseldorf, Germany) set to −20 °C. Before ORO staining, the sections were fixed in 10% formalin for 10 min at room temperature. The liver fragments were stained similarly to the aortic arches, as described above.

The total ORO-stained lesion areas in the aortic arches and fat deposits in the right lobe of the liver were quantified using ImageJ software (version 1.53e) by two independent investigators. The final data were expressed as a percentage of positive-staining areas relative to the total aortic area.

### 2.5. Trichrome Staining

The liver samples were fixed in formalin and dehydrated in an increasing series of alcohols (70%, 80%, 96%, 99.8%), with two changes each for 30 min at room temperature, followed by clearing in two changes of xylene (Warchem, Warsaw, Poland) for 15 min each. After that, the tissues were transferred to a mixture of toluene/paraffin (1:1) and incubated for 2 h at 60 °C. In the final step, each liver lobe was placed in pure molten paraffin overnight and embedded in paraffin blocks. The processed tissue was sectioned into 6 µm thick slices on a microtome (Hyrax M25, Zeiss, Oberkochen, Germany) and placed on microscopic slides. Before staining, the specimens were deparaffinized in two changes of xylene and hydrated in a decreasing series of alcohols. Further, trichrome staining was performed with Trichrome Stain (Masson) Kit (Sigma-Aldrich, St. Louis, MO, USA) according to the manufacturer’s protocol. The total stained areas of fibrosis lesions in the right lobes of the livers were quantified using ImageJ software (version 1.53e) by two independent investigators. Three separate areas of stained lesion were counted and averaged to yield one value per slide. The final data were expressed as a percentage of the positive-staining areas relative to the total aortic area.

### 2.6. Macrophage Immunofluorescence Staining

We performed immunohistochemical staining using F4/80 Monoclonal Antibody (BM8) (Thermo Fisher Scientific, Waltham, MA, USA). The liver lobes were fixed in formalin and dehydrated as described above. After that, the tissues were transferred to a mixture of toluene/paraffin (1:1) and incubated for 2 h at 60 °C. In the final step, each liver lobe was placed in pure molten paraffin overnight and embedded in paraffin blocks. The processed tissue was sectioned into 6 µm thick slices on a microtome (Hyrax M25, Zeiss) and placed on microscopic slides. Before staining, the specimens were deparaffinized in two changes of xylene and hydrated in a decreasing series of alcohols. Heat-induced epitope retrieval was performed at 90 °C using 10 mM Sodium Citrate buffer, pH 6.0, for 20 min. The sections were blocked in 3% BSA. Primary antibodies 1:50 were incubated overnight at 4 °C. The slides were washed in distilled water and mounted with VECTASHIELD Antifade Mounting Medium with DAPI (Vector Laboratories, Inc., Newark, CA, USA). Appropriate positive and negative controls were included with the study sections. The immunostaining was quantified using ImageJ software (version 1.53e) by two independent investigators. Three separate areas of stained lesion were counted and averaged to yield one value per slide. The final data were expressed as a percentage of positive-stained areas relative to the total liver area.

### 2.7. Measurement of mRNA Expression

RNA from the liver lobe was obtained using TRItidy G (PanReac AppliChem, Darmstadt, Germany) according to the manufacturer’s protocol. Total RNA was measured by Nanodrop 2000 (Thermo Fisher Scientific, Waltham, MA, USA). Reverse transcription of RNA was performed using the High-Capacity cDNA Reverse Transcription Kit (Applied Biosystems, Foster City, CA, USA). The expression of *Lpl*, *Srebf1*, and *Fabp4* at the mRNA level in the liver was analyzed using TaqMan probes (Thermo Fisher Scientific) and the TaqMan Real-Time PCR Master Mix (Thermo Fisher Scientific). Reactions were performed on 96-well plates on the LightCycler 96 System (Roche Diagnostics, Germany) Real-Time PCR according to standard protocol. Calculations were made using the LightCycler 96 Software (v.1.1.0.1320). Data were normalized to *Tbp* mRNA levels, and relative quantification was calculated.

### 2.8. Liquid Chromatography–Tandem Mass Spectrometry (LC–MS/MS) Analysis

Mouse livers (*n* = 7 biological replicates per group, in total 14 samples) were homogenized using a Tissue Lyser LT (Qiagen, Hilden, Germany) and lysed in a buffer containing 0.1 M Tris-HCl, pH 7.6, 2% sodium dodecyl sulfate, and 50 mM dithiothreitol (Sigma Aldrich, St. Louis, MO, USA) at 96 °C for 10 min. The total protein concentration in the lysates and the peptide contents in the digests were assayed using a tryptophan fluorescence-based WF assay [29]. Seventy micrograms of protein were digested overnight using the filter-aided sample preparation (FASP) method [30] with Trypsin/Lys-C mix (Promega, Madison, WI, USA) (enzyme-to-protein ratio 1:35) as the digestion enzymes. Next, the samples were purified with C18 Ultra-Micro Spin Columns (Harvard Apparatus, Holliston, MA, USA). All the samples were dissolved in 0.1% formic acid at a concentration of 0.5 µg/µL and spiked with the iRT peptides (Biognosys, Schlieren, Switzerland). One microgram of peptide was injected into a nanoEaseTM M/Z Peptide BEH C18 75 µm i.d. × 25 cm column (Waters, Milford, MA, USA) via a nanoEaseTM M/Z Symmetry C18 180 µm i.d. × 2 cm trap column (Waters, Milford, MA, USA) and separated using a 1% to 40% B phase linear gradient (A phase—0.1% FA in water; B phase—80% ACN and 0.1% FA) with a flow rate of 250 nL/min on an UltiMate 3000 HPLC system (Thermo Scientific, Waltham, MA, USA) coupled to an Orbitrap Exploris 480 Mass Spectrometer (Thermo Scientific, Waltham, MA, USA). The nanoelectrospray ion source parameters were as follows: ion spray voltage: 2.2 kV, ion transfer tube 275 °C. For data-independent (DIA) acquisition, spectra were collected for 145 min in full scan mode (400–1250 Da), followed by 55 DIA scans using a variable precursor isolation window approach and AGC set to a custom 1000%. The DIA MS data were analyzed in Spectronaut 19 (Biognosys, Schlieren, Switzerland) [31] software using the directDIATM approach. MS data were filtered by 1% FDR at the peptide and protein levels, while quantitation was performed at the MS2 level, and global imputation with a missingness rate set to 0.3 was used. Statistical analysis of differential protein abundance was performed at both the MS1 and MS2 levels [32] using unpaired *t*-tests with multiple testing correction after Storey [33]. A summary of the quality control for the LC–MS/MS runs is shown in Supplementary Materials Figure S1. The mass spectrometry data have been deposited in the ProteomeXchange Consortium via the PRIDE partner repository [34] with the dataset identifier PXD063243.

### 2.9. Constructing Protein–Protein Interaction (PPI) Networks

Functional grouping and pathway analysis were performed using PINE (Protein Interaction Network Extractor) software v 1.0.0 [35] with the STRING and GeneMANIA databases using a score confidence of 0.4 and a ClueGO *p* value cutoff < 0.05.

### 2.10. Statistical Analysis

Data collected from serum analysis and histopathological evaluation were checked for normality by the Shapiro–Wilk test. An ANOVA test was utilized to determine any differences between groups, with statistical significance set to *p*-value < 0.05. The results presented on graphs are expressed as means ± SD.

## 3. Results

### 3.1. Effect of the Opioid System Antagonism on the Lipid Profile in Mice with Advanced Atherosclerosis

**Figure 1:**
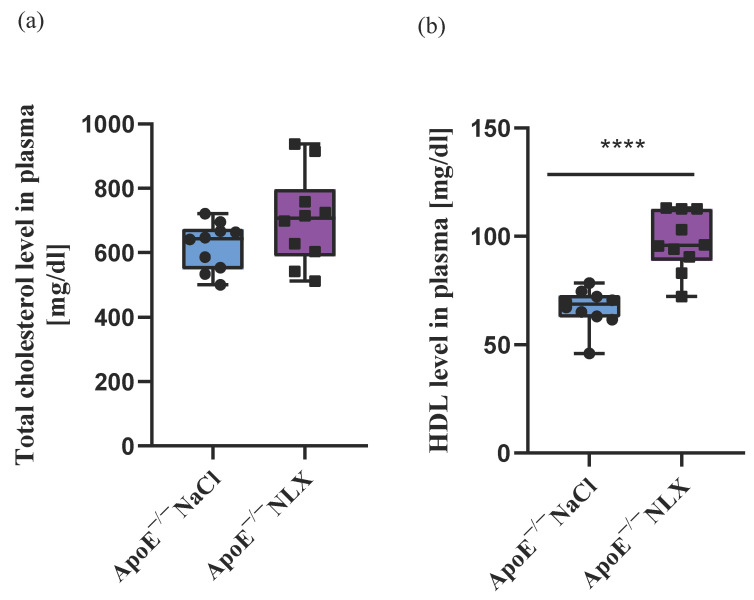

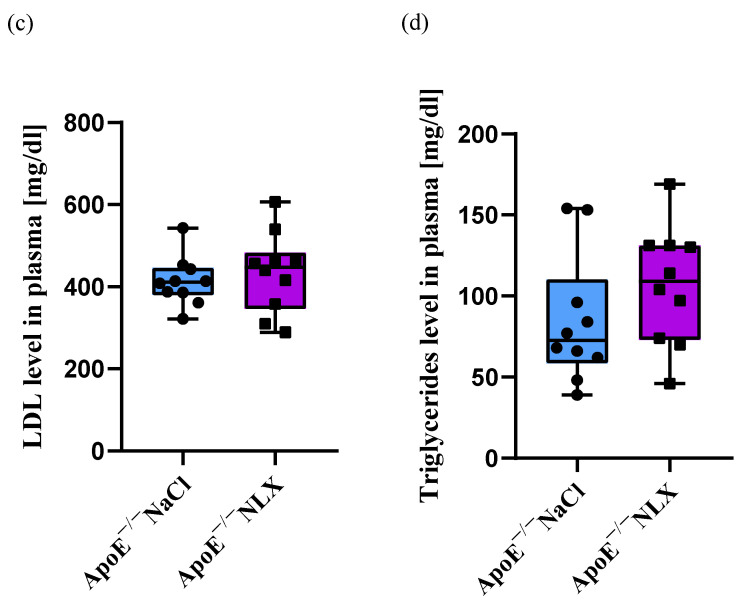
Lipid profile in plasma: (**a**) total cholesterol, (**b**) HDL, (**c**) LDL, (**d**) triglyceride levels in response to administration of NLX in ApoE^−/−^ mice (*n* = 10 per group) **** *p* < 0.0001 (HDL: high-density lipoprotein; LDL: low-density lipoprotein).

Naloxone (NLX) administration did not alter total cholesterol (NaCl 620.90 mg/dL ± 73.36 mg/dL vs. NLX 703.70 mg/dL ± 142.12) [(1, 18) = 2.68; *p* = 0.12], LDL (NaCl 413.10 mg/dl ± 59.40 vs. NLX 434.10 mg/dL ± 97.60) (F[(1, 18) = 0.34; *p* = 0.57]), or triglyceride (NaCl 84.70 mg/dL ± 39.77 vs. NLX 106.60 mg/dL ± 36.30) (F[(1, 18) =1.65; *p* = 0.21]) levels in the ApoE^−/−^ mice (Figure 1). However, the antagonism of the opioid system resulted in elevation of HDL concentration after the NLX treatment (NaCl 66.85 mg/dL ± 9.01 vs. NLX 97.30 mg/dL ± 13.55) (F[(1, 18) =33.00; *p* < 0.001]) (Figure 1).

### 3.2. Effect of the Administration of NLX on Atherosclerotic Plaque and Liver Steatosis in Mice with Advanced Atherosclerosis

**Figure 2:**
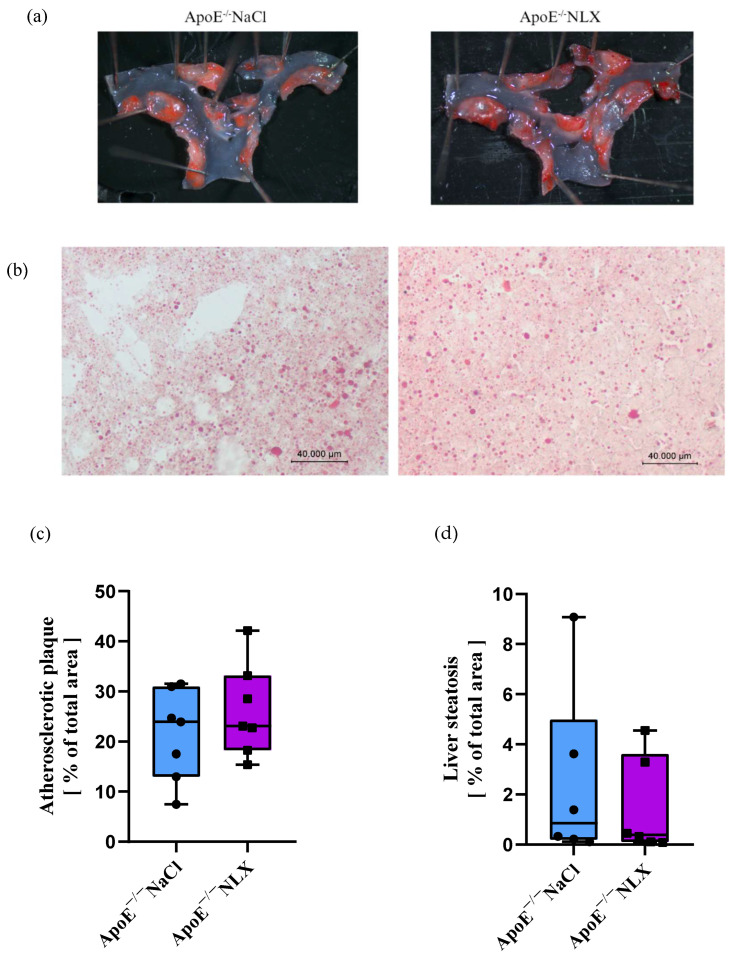
Representative photographs of ORO staining of (**a**) aortic arch (*n* = 7 per group) and (**b**) liver cross-section (microscopic photos performed at 20× magnification) (*n* = 6 per group). Quantified stained area in control and treated groups for (**c**) atherosclerotic plaque and (**d**) liver steatosis.

After 7 days of NLX administration, we did not observe any significant changes in either atherosclerotic plaque localized in aortic arches (NaCl 21.30% ± 9.04 vs. NLX 26.21% ± 9.22) (F[(1, 12) = 1.01; *p* = 0.33]) (Figure 2a) or liver steatosis (NaCl 2.46% ± 3.51 vs. NLX 1.47% ± 1.95) (F[(1, 10) = 0.36; *p* = 0.56]) in the ApoE^−/−^ mice (Figure 2b).

### 3.3. Effect of the NLX Administration on the Expression of Chosen Genes Involved in Lipid Metabolism

**Figure 3:**
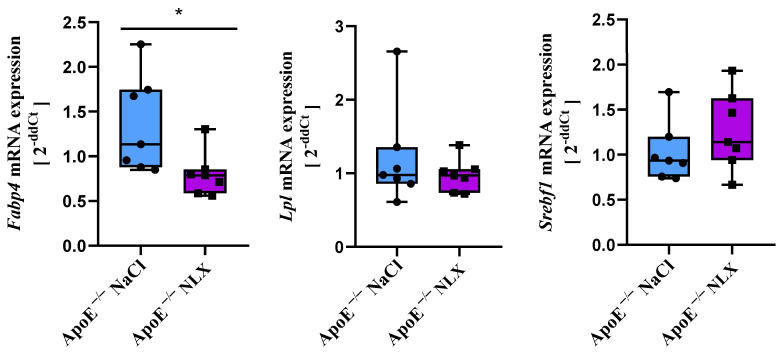
Expression of *Fabp4*, *Lpl*, and *Srebf1* mRNA in livers of ApoE^−/−^ mice (*n* = 7 per group) * *p* < 0.05.

The NLX treatment in the ApoE^−/−^ mice led to a statistically significant decrease in *Fabp4* gene expression (NaCl 1.36 ± 0.54 vs. NLX 0.80 ± 0.25) as shown by significant treatment factor, F[(1, 12) = 6.10; *p* = 0.03]. However, no changes were observed in the expression of the *Lpl* (NaCl 1.09 ± 0.68 vs. 0.98 ± 0.22) (F[(1, 12) = 0.74; *p* = 0.41]) and *Srebf1* (NaCl 1.02 ± 0.33 vs. 1.26 ± 0.43)) (F[(1, 12) = 1.30; *p* = 0.28]) genes (Figure 3).

### 3.4. Influence of NLX Administration on Liver Fibrosis

**Figure 4:**
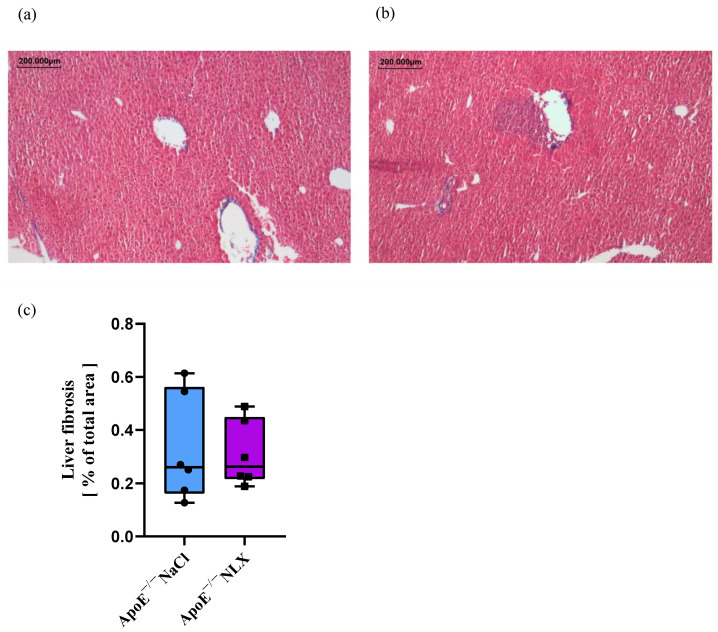
Representative photographs of Masson’s trichrome staining of liver cross-sections in control (**a**) and treated (**b**) groups (microscopic photos performed at 4× magnification) (*n* = 6 per group). Quantified stained area in control and treated (**c**) groups for liver fibrosis.

The NLX administration did not cause statistically significant changes in liver fibrosis (NaCl 0.33% ± 0.20 vs. NLX 0.31% ± 0.12) (F[(1, 10) = 0.04; *p* = 0.84]) (Figure 4).

### 3.5. Effect of the NLX Administration on Macrophage Expression in Mice Liver

**Figure 5:**
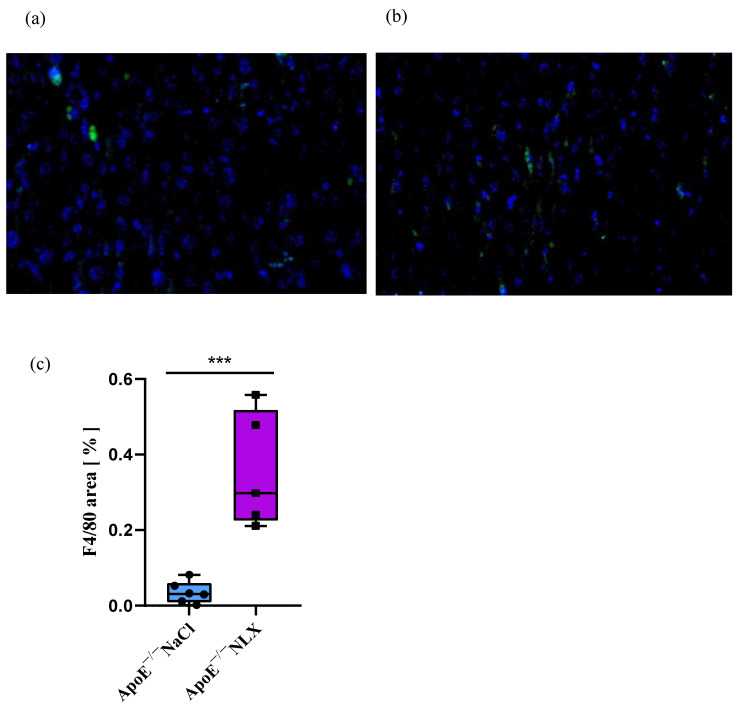
Representative photographs of immunofluorescence staining, with F4/80 antigen as a major macrophage marker, of liver cross-section in control (**a**) and treated (**b**) groups (microscopic photos performed at 20× magnification). Quantified stained area in control and treated groups (**c**) (NaCl *n* = 6, NLX *n* = 5) *** *p* < 0.001.

The NLX treatment caused a statistically significant increase in macrophage expression in comparison to the non-treated group (NaCl 0.04% ± 0.03 vs. NLX 0.36% ± 0.16) (F[(1, 9) = 25.96; *p* < 0.001]) (Figure 5).

### 3.6. Influence of NLX Administration on Proteomic Analysis of Livers

**Figure 6:**
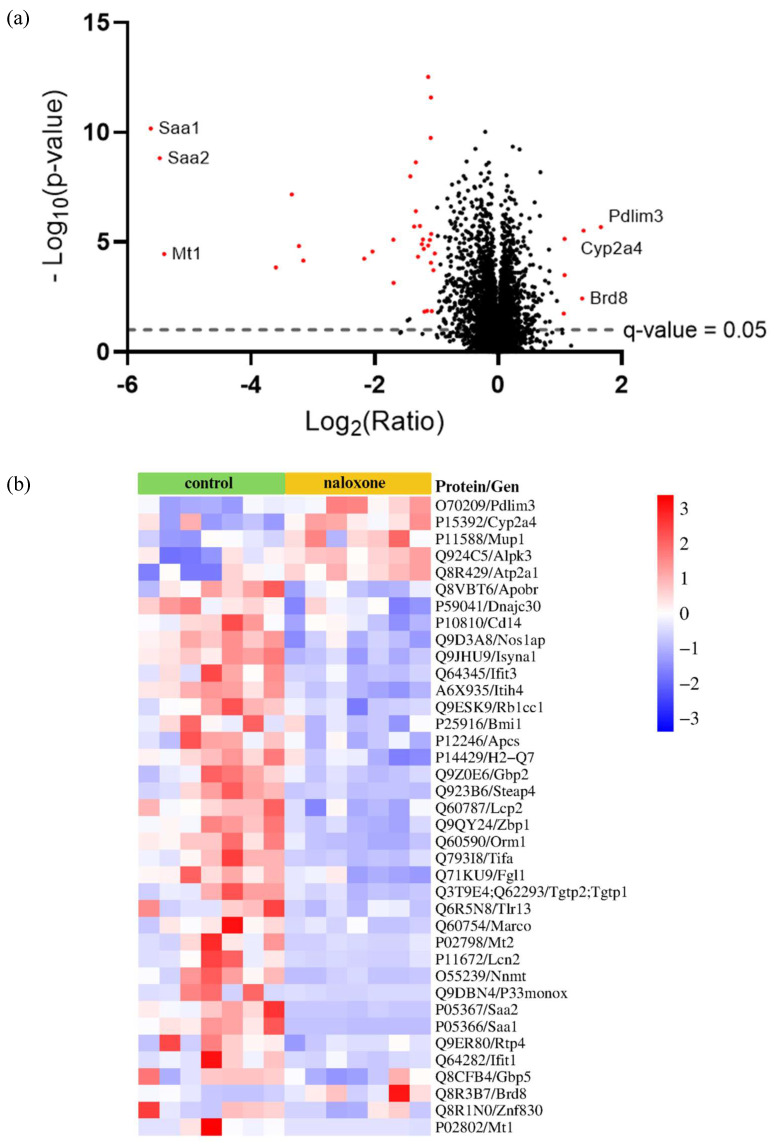

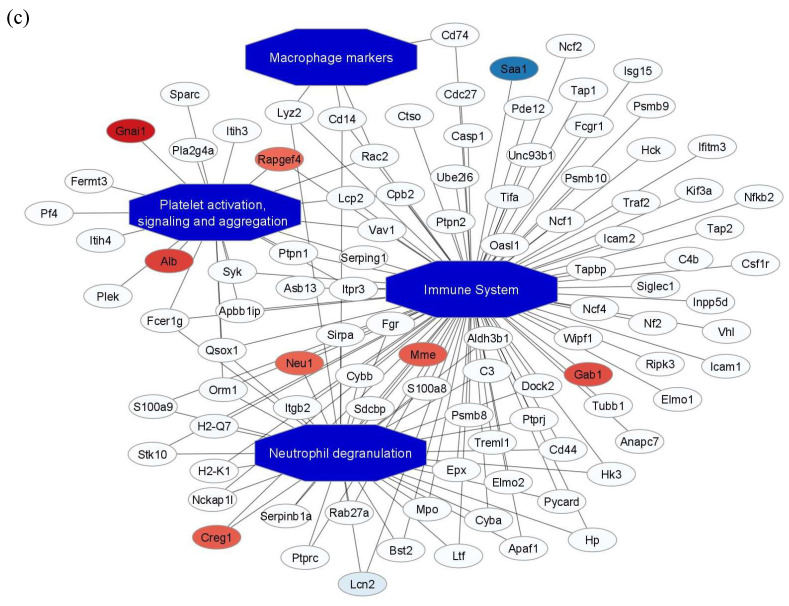
(**a**) Volcano plot for treated and non-treated mice with top six most up- and downregulated proteins shown; (**b**) proteomic data obtained from livers isolated from ApoE^−/−^ mice after NLX administration compared to non-treated (z-score value; cut off; *q* value < 0.05, fold change ≥ 2.0 or ≤−2) (*n* = 7 per group); (**c**) enriched functional network in NTX-treated mice compared to control, as generated by PINE. Inhibited pathways are shown as blue central nodes along with red (upregulated) or blue (downregulated) protein nodes.

To comprehensively investigate the changes in protein abundance upon NLX administration, a quantitative proteomics analysis was conducted. LC–MS/MS measurements operated in DIA mode and analyzed in Spectronaut 19 software with the directDIA approach resulted in the identification of 72,110 proteotypic peptides. In total, 6574 protein groups across all biological conditions were identified and quantified. The median protein group, CV, was approximately 11%, which reflects good reproducibility of data (Supplementary Materials Figure S1D). A summary of the quality control for the LC–MS/MS runs is shown in Supplementary Materials Figure S1. The detailed list of differentially abundant proteins and their fold changes across all the biological conditions is presented in Supplementary Materials Table S1. Using cutoffs of q < 0.05 and fold change ≥ 2 or ≤−2, we found a total of 38 proteins altered in the liver of the NLX-treated mice compared to the control group (Figure 6a–c). This included proteins involved in muscle function and structure (e.g., RB1CC1, PDLIM3), proteins related to cell migration and proliferation (e.g., IFIT3), proteins associated with the inflammatory response and immune system (e.g., GBP, STEAP4, FGL1), proteins involved in cell death (e.g., ZBP1), and proteins that bind heavy metals (e.g., MT1) (Supplementary Materials Table S1).

## 4. Discussion

The endogenous opioid system (EOS) is widely reported in the literature as playing a role in many diseases [24,25,26,27], but its direct involvement in the formation of atherosclerotic plaque and its effect on cholesterol levels are poorly described. Our study demonstrates the effect of blocking opioid receptors with naloxone (NLX) on cholesterol levels and atherosclerotic plaque formation in ApoE^−/−^ mice with advanced atherosclerosis. After 7 days of NLX administration, we did not observe significant changes in the plasma levels of total cholesterol, low-density lipoprotein (LDL), or triglycerides. Our results are comparable to the results shown in other research, where the NLX treatment lasted for 10 weeks [21]. However, we observed a statistically significant increase in the level of high-density lipoprotein (HDL) in plasma after the NLX administration. Interestingly, a cross-sectional study of 2239 opioid users found that opioid use was associated with significantly lower levels of total cholesterol, LDL, and HDL [34,35].

Moreover, several reports indicate that an opioid receptor antagonist affects cholesterol levels. It has been shown that a 5-day pretreatment with naltrexone prevented stress-induced increases in cholesterol levels in female rats fed a cholesterol–cholic acid (CCA)-supplemented diet [24]. Moreover, in the same animal model, a 5-day release of 75 mg of morphine elevated total plasma cholesterol and LDL, reduced HDL, and increased cholesterol deposition in the aorta. However, the daily administration of 1 mg/kg naltrexone completely reversed these effects on both cholesterol levels and aortic cholesterol deposition [36].

Due to the role of the liver in regulating lipid metabolism, it may be a key factor in the processes related to the development of atherosclerosis. On the other hand, the progression of atherosclerosis contributes to pathological liver changes, such as steatosis [37,38]. This is supported by studies showing that patients with liver steatosis have significantly higher levels of LDL and lower levels of HDL, compared to those without liver steatosis [39]. Impaired HDL function has been postulated to be an independent risk factor for atherosclerosis in non-alcoholic fatty liver disease [40]. Furthermore, research indicates that liver steatosis is more prevalent in patients with carotid plaques and aortic calcification than in those with no vascular damage [41]. A study on children and adolescents revealed an association between increased intima–media thickness of the carotid artery, a high triglyceride/HDL-C ratio, elevated blood pressure, insulin resistance, and non-alcoholic fatty liver disease [42]. Although we observed a significant increase in plasma HDL levels following the NLX treatment, the functional relevance of this change remains to be determined. Recent studies emphasize that HDL cholesterol levels alone do not fully reflect their atheroprotective functions, such as cholesterol efflux capacity (CEC), antioxidant properties, or anti-inflammatory effects [43]. Therefore, it is possible that the HDL elevation observed in our model may not necessarily confer a cardioprotective benefit. In fact, increased levels of serum amyloid A (SAA)—which was downregulated in our study after naloxone treatment—have been shown to affect cholesterol efflux by displacing apoA-I from HDL particles, thereby impairing HDL function. This suggests that the observed decrease in SAA protein abundance may contribute to improved HDL functionality, although this hypothesis requires further experimental validation.

In our study, we also investigated the effects of opioid receptor blockade on atherosclerotic plaque development and liver steatosis in mice. We observed neither significant changes in the development of atherosclerotic plaques nor fat deposits in the liver after the NLX treatment. However, one study showed a significant reduction in atherosclerotic plaque after long-term, 10-week administration of NLX in 17-week-old mice. Moreover, the study demonstrated that naloxone inhibits macrophage activation induced by LPS (lipopolysaccharide) and oxidized LDL (oxLDL). Naloxone treatment reduced TNF-α production and superoxide production in stimulated macrophages [21]. This may indicate that prolonged administration of NLX at an early stage of atherosclerotic plaque development, rather than during the advanced stages observed in our studies, may significantly reduce plaque development and progression. Similarly, while we did not observe changes in liver steatosis, other studies have shown that long-term administration of NLX can reverse morphine-induced histopathological damage [44]. Interestingly, the administration of another opioid receptor antagonist, naltrexone, for 28 days, in BDL rats (rats with ligated bile ducts, used as a model of liver fibrosis) [45] significantly attenuates the development of liver fibrosis [27]. NLX, as an opioid system antagonist, by definition, has no intrinsic pharmacological activity apart from the ability to bind to opioid receptors and simply block them. It reveals a significant effect in the form of antagonizing opioid agonists; therefore, its effect, when applied in clinically therapeutic and safe doses, may not be visible without agonist-mediated opioid receptor activation [46].

Although histological analysis did not reveal changes in hepatic steatosis after NLX administration, we investigated the expression of genes associated with its development. The first gene we examined was *Srebf1*. This gene encodes Sterol Regulatory Element-Binding Transcription Factor 1 (SREBF1), which is a key factor in lipogenesis. Lipogenesis is a metabolic pathway that converts excess carbohydrates to fatty acids, which are ultimately esterified with glycerol-3-phosphate to triglycerides [47]. The important role of *Srebf1* in lipogenesis is confirmed by studies indicating that the expression of this gene in the livers of patients with non-alcoholic steatosis is significantly higher than in healthy individuals [48]. Moreover, the reduced expression of the *Sreb-1c* gene in hepatocytes by activating 5′AMP-activated protein kinase (AMPK) protected against hepatic steatosis and hyperlipidemia in diet-induced insulin-resistant LDL receptor-deficient mice [49]. After the NLX administration, we did not observe changes in *Srebf1* expression in the livers of the ApoE^−/−^ mice. The next gene we investigated was *Lpl*. It encodes an enzyme crucial for the hydrolysis of core triglycerides in chylomicrons and very low-density lipoproteins (VLDL), resulting in the formation of chylomicron remnants and intermediate-density lipoproteins (IDL), respectively. The reaction products catalyzed by LPL, fatty acids, and monoacylglycerol are partially absorbed by local tissues and processed. For instance, they are stored as neutral lipids in adipose tissue, oxidized or stored in skeletal and cardiac muscle, or deposited as cholesterol esters and triglycerides in macrophages [50,51]. Research indicates that patients with severe liver steatosis exhibit significantly higher serum LPL activity compared to those with mild or moderate steatosis [52]. Moreover, morbidly obese individuals show statistically increased hepatic LPL activity compared to controls, independent of liver fibrosis or fatty liver presence [53]. However, in our study, after treatment with NLX, we did not observe any changes in *Lpl* mRNA expression in the liver. We also investigated the expression of the *Fabp4* gene. It encodes a small, highly conserved cytosolic protein that binds long-chain fatty acids and other hydrophobic ligands, regulating lipid transport within the cell [54]. It is primarily found in adipose tissue but is also detected in macrophages, endothelial cells, and cardiac myocytes [54,55]. Studies have shown that the use of an Fabp4 inhibitor, at a dose of 50 mg/kg/day for 8 weeks, in combination with rosiglitazone (a drug used in type II diabetes, which acts as an insulin sensitizer), resulted in a reduction of rosiglitazone-induced hepatic steatosis in obese diabetic C57BL/KsJ *db/db* mice [56]. In our study, after the NLX treatment, we did not observe changes in hepatic steatosis in the histological examination; however, at the molecular level, we observed a significant decrease in *Fabp4* mRNA expression in the livers of the ApoE^−/−^ mice (fold change 0.8). FABP4 plays a crucial role in the transport, metabolism, and storage of lipids. Elevated levels of FABP4 have been associated with liver steatosis, particularly in individuals with metabolic conditions like diabetes and obesity [57]. Therefore, we believe that the changes in *Fabp4* mRNA expression we observed are indicators of the initiation of fat accumulation in the liver, which was not yet visible at the histological level. Moreover, liver fibrosis, as one of the indicators of liver steatosis, was not altered after naloxone administration.

In rats with dimethylnitrosamine-induced liver fibrosis, naloxone reduced collagen deposition after five weeks of treatment [58]. Additionally, naltrexone significantly attenuated the progression of liver fibrosis in bile duct-ligated rats [27]. However, in the mentioned studies, naloxone was administered for a relatively longer period than in our study. Another marker indicating liver steatosis is an increased level of macrophages in the liver.

We evaluated the presence of macrophages in the livers of ApoE^−/−^ mice following NLX administration. A 7-day NLX treatment increased the macrophage population compared to the control group. It is known that macrophages increase their number in the liver with steatosis. However, macrophages are highly heterogeneous cells with diverse physiological and pathophysiological functions, exhibiting both pro-atherogenic (M1) and protective (M2) effects [59,60]. We have not evaluated the subset of macrophages in the liver after naloxone treatment, which is a limitation of the study. Experiments performed by Liu et al. show that naloxone pretreatment significantly suppressed the production of tumor necrosis factor-alpha (TNF-alpha), interleukin-6, monocyte chemoattractant protein-1, and superoxide in macrophages after stimulation [23]. A similar effect was observed in murine RAW264.7 cells, showing an anti-inflammatory effect of naloxone on macrophages [61].

In our study, the most significant effect of the NLX was observed on HDL levels. Since the liver is an important site of cholesterol metabolism, we performed a proteomic analysis of the liver in ApoE^−/−^ mice treated with naloxone. The analysis detected 6574 proteins, of which 38 were significantly up- or downregulated. Then, we selected four proteins, whose abundance levels were significantly altered after NLX administration and which influence the lipid profile and are involved in the development of atherosclerosis. The first of them is apolipoprotein B receptor (Q8VBT6) encoded by the *Apobr* gene, whose abundance decreased after the NLX treatment. It is a receptor for apolipoprotein B (ApoB), which plays a crucial role in the development of atherosclerosis [62]. Studies have also shown that the use of an ApoB inhibitor causes a decrease in the level of total cholesterol and LDL in mice [63] and the level of LDL in humans [64]. In our study, NLX treatment reduced the abundance of apolipoprotein B receptor. The next protein downregulated by NLX that is worth mentioning is serum amyloid A (SAA) (P05367), encoded by the *Saa2* gene. SAA is a small apolipoprotein that binds to HDLs. SAA is an acute-phase marker and inflammation factor, with its levels significantly elevated in human serum during chronic inflammation [65,66]. Additionally, studies have shown that in patients with endotoxin-induced inflammation, the ability of macrophages to efflux HDL correlates with increased levels of SAA1 and SAA2. In mice, acute inflammation similarly leads to a marked impairment in HDL efflux, accompanied by a substantial rise in SAA levels [67]. Another protein closely associated with cholesterol levels, whose abundance was altered in our study by NLX, is nicotinamide N-methyltransferase (O55239) encoded by the *Nnmt* gene. The abundance of this protein was significantly reduced after the NLX treatment [68,69]. Studies have also demonstrated that inhibition of NNMT in obese mice decreased total cholesterol levels in plasma [70], and knock-out of the *Nnmt* gene led to lower total cholesterol and LDL levels in mice with non-alcoholic steatohepatitis [71]. Another protein which plays a role in lipid metabolism and whose abundance was changed by NLX is major urinary protein 1 (P11588), encoded by the *Mup1* gene. Following NLX administration, we observed an increase in MUP1 protein abundance. MUP1 is a lipocalin family member abundantly secreted into the circulation by the liver [72]. Except for its basic role, which is binding to lipophilic pheromones to support chemical communication in rodents, MUP1 regulates glucose and lipid metabolism. What is worth noting is that MUP1 inhibits lipogenesis. Hepatic high-abundance of MUP1 suppressed the abundance of stearoyl-CoA desaturase-1 (SCD1), fatty acid synthase (FAS), carbohydrate response element-binding protein (ChREBP), and peroxisome proliferator-activated receptor-γ (PPARγ) in *db/db* mice [73].

## 5. Conclusions

In summary, the above results indicate a connection between the opioid system, cholesterol levels, and the development of atherosclerosis. However, our 7-day NLX administration model in mice with advanced atherosclerosis appears insufficient to demonstrate a direct effect of opioid receptor blockade on lipid profiles and atherosclerotic plaque development. It would be beneficial to conduct further studies with an extended treatment duration or in a model where atherosclerosis is just beginning to develop. It would also be worthwhile to conduct a proteomic study of blood vessel samples and examine the effect of local naloxone administration on the liver or the atherosclerotic vessel [74]. This would provide more information about the direct role of naloxone on the studied organs.

## Data Availability

The original data presented in the study are openly available to the ProteomeXchange Consortium via the PRIDE partner repository with the dataset identifier PXD063243.

## Supplementary Materials

The following supporting information can be downloaded at: https://www.mdpi.com/article/10.3390/biomedicines13081802/s1, Figure S1: Quality control of MS runs of the liver of control and naloxone-treated mice; Table S1: An overview of deregulated proteins, their encoding genes, and functions. References [75,76,77,78,79,80,81,82,83,84,85,86,87,88,89,90,91,92,93,94,95,96,97,98,99,100,101,102,103,104,105,106,107,108,109] are cited in Supplementary Materials.
