# Supplementary material for: Targeting the Opioid System in Cardiovascular Disease: Liver Proteomic and Lipid Profile Effects of Naloxone in Atherosclerosis
Source: Biomedicines. 2025 Jul 23;13(8):1802. doi: 10.3390/biomedicines13081802 (PMC12383304; doi:10.3390/biomedicines13081802)
Supplement: Supplementary file 1 [file biomedicines-13-01802-s001.zip › biomedicines-3749593-supplementary.pdf]

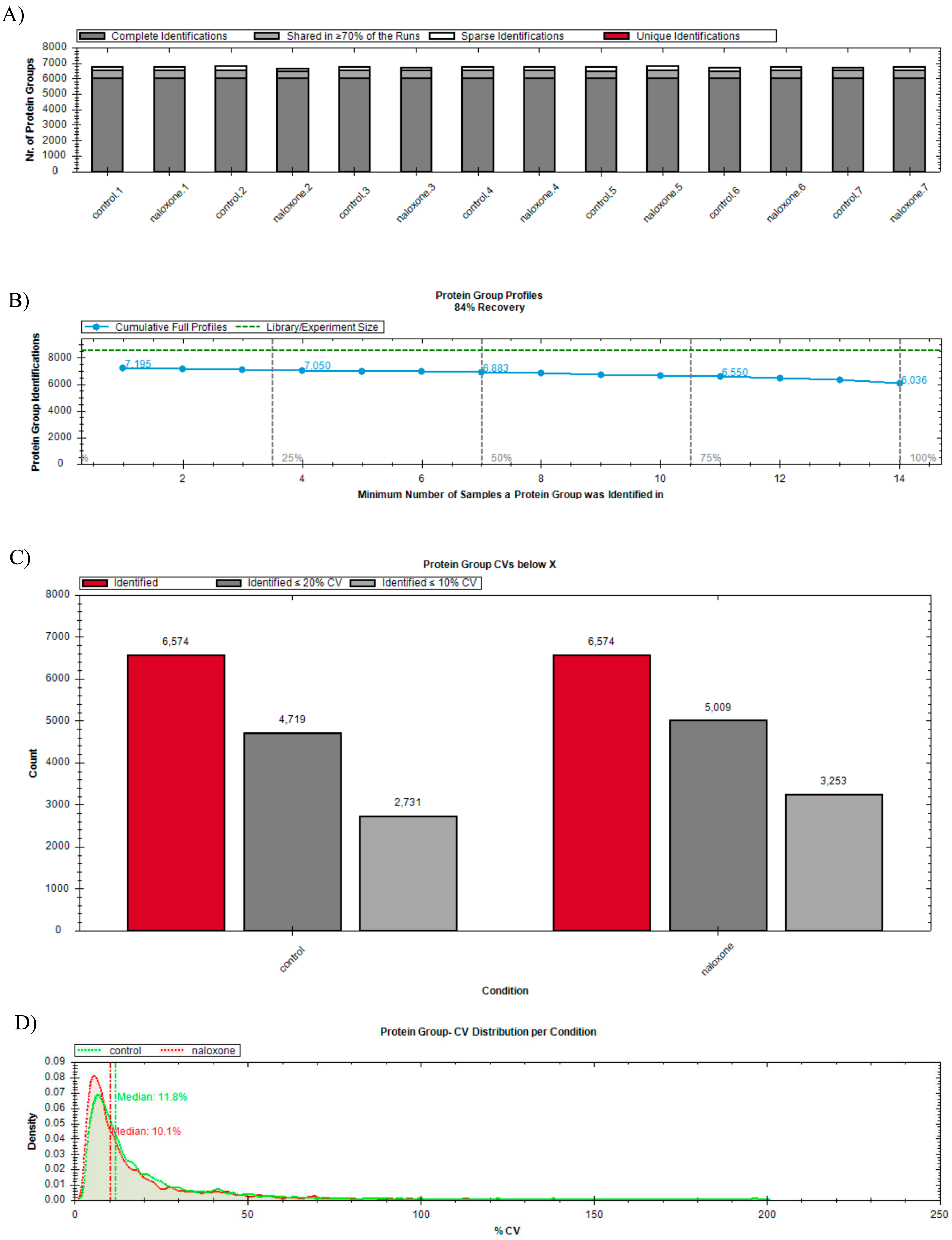

Figure 1. Quality control of MS runs of the liver of control and naloxone-treated mice. Protein group identification details across all LC-MS runs **(A)**. Spectral library recovery **(B)**. Coefficient of variations (CVs) for protein groups across all biological conditions **(C)**. Distribution of protein group CV in biological conditions **(D)**(n = 7 biological replicates).

Table 1. An overview of deregulated proteins, their encoding genes, and functions (functions obtained from UniProt database [1]).

Protein	Gene	Fold change	Main function	Function connected to lipid metabolism and atherosclerosis
O70209	*Pdlim3* (PDZ and LIM domain 3)	3.16	May play a role in the organization of actin filament arrays within muscle cells.	Upregulated in patients with familiar hypercholesterolemia (FH) or with FH-related coronary heart disease [2].
P15392	*Cyp2a4* (liver cytochrome 2a4)	2.60	Highly active in the 15-alpha-hydroxylation of testosterone.	Upregulated in $ApoE^{-/-}$ mice overexpressing proline/serine-rich coiled-coil 1 (PSRC1) that regulates blood lipid levels and inhibit atherosclerosis [3].
Q8R3B7	*Brd8* (bromo-conatining protein 8)	2.56	May act as a coactivator during transcriptional activation by hormone-activated nuclear receptors.	Brd8/p400 (p400) chromatin remodeling complex promotes adipogenesis [4].
P11588	*Mup1* (major urinary protein 1)	2.10	Binds pheromones that are released from drying urine of males.	Belong to lipocalins family that transport hydrophobic molecules as steroids, retinoids, lipids [5]. Significantly reduced in genetic and dietary fat-induced obesity and diabetes in bd/bd mice, while administration of recombinant MUP1 attenuated hyperglycemia and glucose intolerance in these mice [6].
Q924C5	*Alpk3* (alpha kinase 3)	2.10	Involved in cardiomyocyte differentiation.	ALPK3-deficient mice develop cardiac hypertrophy [7].
Q8R429	*Atp2a1* (ATPase sarcoplasmic/endoplasmic reticulum Ca^{2+} transporting 1)	2.09	Contributes to calcium sequestration involved in muscular excitation/contraction.	Atp2a1 is significantly increased in male offspring of murine mothers fed high linoleic acid diet but unchanged in female offspring [8].
Q8CFB4	*Gbp5* (Guanylate binding protein 5)	-2.04	Plays important roles in innate immunity against a diverse range of bacterial, viral and protozoan pathogens.	suggested as a potential atherosclerosis marker involving in inflammation [9,10].
Q8VBT6	*Apobr* (apolipoprotein B receptor)	-2.07	May provide essential lipids to reticuloendothelial cells. Could also be involved in foam cell formation with elevated triglyceride-rich lipoproteins and remnant lipoprotein.	-
P59041	*Dnajc30* (DnaJ heat shock protein family (Hsp40) member C30)	-2.11	Mitochondrial protein enriched in neurons that acts as a regulator of mitochondrial respiration	-

P10810	*Cd14*<br>(cluster of differentiation 14)	-2.12	Expressed by macrophages, mediating the innate immune response to bacterial lipopolysaccharide.	Cd14 is involved in pathways contributing to worsening of atherosclerosis. *Cd14* knockdown blocks oxidized LDL uptake in macrophages and foam cell formation in fatty deposits in blood vessels [11].
Q9D3A8	*Nos1ap*<br>(nitric oxide synthase 1 adaptor protein)	-2.13	Adapter protein involved in neuronal nitric-oxide (NO) synthesis.	Liver specific NOS1AP conditional knockout mice demonstrated increased lipid deposits in the liver [12].
Q9ER80	*Rtp4*<br>(receptor transporter protein 4)	-2.13	Promotes functional expression of the opioid receptor heterodimer OPRD1-OPRM1. Probable chaperone protein which facilitates trafficking and functional cell surface expression of some G-protein coupled receptors.	Morphine administration to C57BL/6 mice increased Rtp4 mRNA expression in the hypothalamus as well as caused a significant increase in μ-δ opioid receptor heterodimers and μ-opioid receptors. RTP4 is necessary and sufficient for the regulation of opioid receptor presence on the cell surface [13]<br>RTP4 protects mu-delta receptors from ubiquitination and degradation [14].
Q9JHU9	*Isyna1*<br>(inositol-3-phosphate synthase 1)	-2.14	Key enzyme in myo-inositol biosynthesis pathway that catalyzes the conversion of glucose 6-phosphate to 1-myo-inositol 1-phosphate in a NAD-dependent manner.	-
Q64345	*Ifit3*<br>(interferon induced protein with tetratricopeptide repeats 3)	-2.15	Acts as an inhibitor of cellular as well as viral processes, cell migration, proliferation, signaling, and viral replication.	Analysis on the mRNA expression profile of human atherosclerotic samples obtained from GEO database identified IFIH1, IFIT1, IFIT2, IFIT3, ISG15 and OAS3 as immune-related hub genes of atherosclerosis [15].
A6X935	*Itih4*<br>(inter-alpha-trypsin inhibitor heavy chain 4)	-2.20	Type II acute-phase protein (APP) involved in inflammatory responses to trauma.	Itih4 has been identified as a smooth muscle cell-expressed gene in atherosclerotic plaques [16].<br>Genetic variation (SNP) in human *Itih4* gene determines cholesterol metabolism and its level in blood plasma [17].
Q9ESK9	*Rb1cc1*<br>(RB1-inducible coiled-coil protein 1)	-2.20	Involved in autophagy. Plays a crucial role in muscular differentiation.	In Rb1cc1-CKO mice increased accumulation of lipid-filled vacuoles in the retinal pigment epithelium was observed [18].
Q8R1N0	*Znf830*<br>(zinc finger protein 830)	-2.22	May play a role in pre-mRNA splicing as component of the spliceosome	-
P25916	*Bmi1*	-2.29	Component of a Polycomb group (PcG) multiprotein PRC1-like complex, a complex class required to	-

	(polycomb complex protein BMI-1)		maintain the transcriptionally repressive state of many genes.	
P12246	*Apcs* (serum amyloid P-component)	-2.31	Can interact with DNA and histones and may scavenge nuclear material released from damaged circulating cells.	-
P14429	*H2-Q7* (H-2 class I histocompatibility antigen, Q7 alpha chain)	-2.33	Involved in the presentation of foreign antigens to the immune system.	-
Q9Z0E6	*Gbp2* (Guanylate-binding protein 2)	-2.36	Interferon (IFN)-inducible GTPase that plays important roles in innate immunity against a diverse range of bacterial, viral and protozoan pathogens.	*Gbp2* expression was upregulated in mouse peritoneal macrophage culture treated with oxLDL [19].
Q923B6	*Steap4* (metalloreductase STEAP4)	-2.41	Plays a role in systemic metabolic homeostasis, integrating inflammatory and metabolic responses. Involved in inflammatory arthritis, through the regulation of inflammatory cytokines.	Diet-induced obesity caused a significant increase in STEAP4 mRNA expression in rats PBMCs [20].
Q60787	*Lcp2* (lymphocyte cytosolic protein 2)	-2.46	Involved in T-cell antigen receptor mediated signaling	LCP2 was found, among others, to be connected with carotid atherosclerotic plaques [21]. Analysis of aortas of $ApoE^{-/-}$ mice fed a Western diet for 6 months demonstrated high expression of LCP2 as compared to the control group [22].
Q9QY24	*Zbp1* (Z-DNA-binding protein 1)	-2.52	Key innate sensor that recognizes and binds Z-RNA structures, which are produced by a number of viruses, and triggers different forms of cell death.)	Deletion of ZBP1 substantially decreases the steatotic liver ischemia/reperfusion injury [23]. *Zbp1* knockout mice treated with LDLr antisense oligos and fed a western-type diet demonstrated tendency to a decrease in median aortic lesion area compared to WT group. Also, median necrotic lesion area and % necrotic lesion area were decreased [24].
Q60590	*Orm1* (alpha-1-acid glycoprotein 1)	-2.52	Functions as a transport protein in the blood stream.	-
Q793I8	*Tifa* (TNF-α receptor-associated factor-interacting protein with a forkhead-associated domain)	-2.57	Adapter molecule that plays a key role in the activation of pro-inflammatory NF-kappa-B signaling following detection of bacterial pathogen-associated molecular pattern metabolites (PAMPs).	TIFA protein levels were significantly increased in the aortas of $ApoE^{-/-}$ mice receiving a Western diet compared to the chow diet; furthermore, oxidized LDL treatment increased *Tifa* mRNA and protein levels in cultured human umbilical vein endothelial cells [25].

Q71KU9	*Fgl1* (fibrinogen-like protein 1)	-2.68	Immune suppressive molecule that inhibits antigen-specific T-cell activation by acting as a major ligand of LAG3. Secreted by, and promotes growth of, hepatocytes	Liver injury enhance the expression of *Fgl1* in brown adipose tissue. Fgl1 null mice have greater body mass and abnormal plasma lipid profiles than wild type mice [26].
Q64282	*Ifit1* (Interferon-induced protein with tetratricopeptide repeats 1)	-3.24	Interferon-induced antiviral RNA-binding protein, acting as a sensor of viral single-stranded RNAs and inhibiting expression of viral messenger RNAs.	Based on Gene Expression Omnibus database IFIT1 has been identified as associated with atherosclerosis [15]. IFIT1 together with IFIT3 have been evidenced to be involved in pro-inflammatory polarization of macrophages and decreased collagen deposition leading to atherosclerotic plaque vulnerability [27].
Q3T9E4; Q62293	*Tgtp2; Tgtp1* (T-cell-specific guanine nucleotide triphosphate-binding protein 2;-1)	-3.25	Involved in innate cell-autonomous resistance to intracellular pathogens such as Toxoplasma gondii; Involved in innate cell-autonomous resistance to intracellular pathogens, such as Toxoplasma gondii.	-
Q6R5N8	*Tlr13* (Toll-like receptor 13)	-4.10	Component of innate and adaptive immunity that recognizes and binds 23S rRNA from bacteria. TLRs (Toll-like receptors) control host immune response against pathogens.	-
Q60754	*Marco* (macrophage receptor with collagenous structure)	-4.50	Pattern recognition receptor (PRR) which binds Gram-positive and Gram-negative bacteria.	Macrophage receptor with collagenous structure (MARCO) are membrane glycoproteins mediating the uptake of chemically modified low density lipoproteins [28].
P02798	*Mt2* (metallothionein-2)	-8.92	Metallothioneins have a high content of cysteine residues that bind various heavy metals.	-
P11672	*Lcn2* (lipocalin 2)	-9.37	Iron-trafficking protein involved in multiple processes such as apoptosis, innate immunity and renal development.	*Lcn2* knockout mice developed larger atherosclerotic lesions during earlier stages of atherosclerosis compared to control [29]. Chronic administration of LCN2 in ApoE$^{-/-}$ mice markedly accelerated the development of aortic atherosclerotic lesions and increased lesion infiltration with monocyte/macrophage [30]. Overexpression of human Lcn2 in hepatocytes attenuates the development of atherosclerosis in western diet-fed Ldlr$^{-/-}$ mice [31].

O55239	*Nnmt* (nicotinamide N-methyltransferase)	-10.15	Acts as a metabolic regulator primarily on white adipose tissue energy expenditure as well as hepatic gluconeogenesis and cholesterol biosynthesis.	NNMT plays an important role in hepatic detoxification. Product of the NNMT catalysis is homocysteine which is considered a marker of cardiovascular disease, probably acting via atherogenesis [32].<br>Inhibition of hepatic *Nnmt* expression in vivo affects cholesterol metabolism [33].
Q9DBN4	*P33-monox* (putative monooxygenase p33MONOX)	-12.16	Potential NADPH-dependent oxidoreductase. May be involved in the regulation of neuronal survival, differentiation and axonal outgrowth.	-
P02802	*Mt1* (metallothionein 1)	-42.61	Metallothioneins have a high content of cysteine residues that bind various heavy metals; these proteins are transcriptionally regulated by both heavy metals and glucocorticoids.	-
P05367	*Saa2* (serum amyloid A2)	-44.70	Major acute phase reactant. Apolipoprotein of the HDL complex	Absence of SAA1 and SAA2 attenuated angiotensin-induced abdominal aneurysm formation in $ApoE^{-/-}$ mice [34].
P05366	*Saa1* (serum amyloid A1)	-49.41	Major acute phase reactant	$ApoE^{-/-}$ mice overexpressed murine SAA1 exhibited modest but persistent increase in SAA that contributed to increased atherosclerosis via increased inflammatory cell infiltration [35].<br>$Rag1^{-/-}ApoE^{-/-}$ and $ApoE^{-/-}$ mice injected with adenoviral vector encoding human SAA1 had increased atherosclerosis compared with controls. It was also established that SAA treatment contribute to increased LDL retention in vascular smooth muscle cell culture [36].

[1] UniProt: the Universal Protein Knowledgebase in 2023. Nucleic Acids Res 2023;51:D523–31. https://doi.org/10.1093/nar/gkac1052.

[2] Prasongsukarn K, Dechkhajorn W, Benjathummarak S, Maneerat Y. TRPM2, PDLIM5, BCL3, CD14, GBA Genes as Feasible Markers for Premature Coronary Heart Disease Risk. Front Genet 2021;12. https://doi.org/10.3389/FGENE.2021.598296.

[3] Wei M, Li P, Guo K. The impact of PSRC1 overexpression on gene and transcript expression profiling in the livers of ApoE-/- mice fed a high-fat diet. Mol Cell Biochem 2020;465:125–39. https://doi.org/10.1007/S11010-019-03673-X.

[4] Couture JP, Nolet G, Beaulieu E, Blouin R, Gévry N. The p400/Brd8 chromatin remodeling complex promotes adipogenesis by incorporating histone variant H2A.Z at PPARγ target genes. Endocrinology 2012;153:5796–808. https://doi.org/10.1210/EN.2012-1380.

[5] Greve S, Kuhn GA, Saenz-de-Juano MD, Ghosh A, von Meyenn F, Giller K. The major urinary protein gene cluster knockout mouse as a novel model for translational metabolism research. Sci Rep 2022;12:13161. https://doi.org/10.1038/S41598-022-17195-Y.

[6] Zhou Y, Jiang L, Rui L. Identification of MUP1 as a Regulator for Glucose and Lipid Metabolism in Mice. Journal of Biological Chemistry 2009;284:11152–9. https://doi.org/10.1074/JBC.M900754200.

[7] Van Sligtenhorst I, Ding Z-M, Shi Z-Z, Read RW, Hansen G, Vogel P. Cardiomyopathy in α-kinase 3 (ALPK3)-deficient mice. Vet Pathol 2012;49:131–41. https://doi.org/10.1177/0300985811402841.

[8] Shrestha N, Sleep S, Helman T, Holland O, Cuffe JSM, Perkins A V., Mcainch AJ, Headrick JP, Hryciw DH. Maternal diet high in linoleic acid alters offspring fatty acids and cardiovascular function in a rat model. Br J Nutr 2022;127:540–53. https://doi.org/10.1017/S0007114521001276.

[9] Shenoy AR, Wellington DA, Kumar P, Kassa H, Booth CJ, Cresswell P, MacMicking JD. GBP5 promotes NLRP3 inflammasome assembly and immunity in mammals. Science 2012;336:481–5. https://doi.org/10.1126/SCIENCE.1217141.

[10] Chmielewski S, Olejnik A, Sikorski K, Pelisek J, Błaszczyk K, Aoqui C, Nowicka H, Zernecke A, Heemann U, Wesoly J, et al. STAT1-dependent signal integration between IFNγ and TLR4 in vascular cells reflect pro-atherogenic responses in human atherosclerosis. PLoS One 2014;9. https://doi.org/10.1371/JOURNAL.PONE.0113318.

[11] An D, Hao F, Zhang F, Kong W, Chun J, Xu X, Cui MZ. CD14 is a key mediator of both lysophosphatidic acid and lipopolysaccharide induction of foam cell formation. Journal of Biological Chemistry 2017;292:14391–400. https://doi.org/10.1074/JBC.M117.781807.

[12] Mu K, Sun Y, Zhao Y, Zhao T, Li Q, Zhang M, Li H, Zhang R, Hu C, Wang C, et al. Hepatic nitric oxide synthase 1 adaptor protein regulates glucose homeostasis and hepatic insulin sensitivity in obese mice depending on its PDZ binding domain. EBioMedicine 2019;47:352–64. https://doi.org/10.1016/J.EBIOM.2019.08.033.

[13] Fujita W, Yokote M, Gomes I, Gupta A, Ueda H, Devi LA. Regulation of an Opioid Receptor Chaperone Protein, RTP4, by Morphine s. MOLECULAR PHARMACOLOGY Mol Pharmacol 2019;95:11–9. https://doi.org/10.1124/mol.118.112987.

[14] Décaillot FM, Rozenfeld R, Gupta A, Devi LA. Cell surface targeting of mu-delta opioid receptor heterodimers by RTP4. Proc Natl Acad Sci U S A 2008;105:16045–50. https://doi.org/10.1073/PNAS.0804106105.

[15] Dong R, Jiang G, Tian Y, Shi X. Identification of immune-related biomarkers and construction of regulatory network in patients with atherosclerosis. BMC Med Genomics 2022;15. https://doi.org/10.1186/S12920-022-01397-4.

[16] Ravindran A, Holappa L, Niskanen H, Skovorodkin I, Kaisto S, Beter M, Kiema M, Selvarajan I, Nurminen V, Aavik E, et al. Translatome profiling reveals Itih4 as a novel smooth muscle cell-specific gene in atherosclerosis. Cardiovasc Res 2024;120:869–82. https://doi.org/10.1093/cvr/cvae028.

[17] Fujita Y, Ezura Y, Emi M, Sato K, Takada D, Iino Y, Katayama Y, Takahashi K, Kamimura K, Bujo H, et al. Hypercholesterolemia associated with splice-junction variation of inter-alpha-trypsin inhibitor heavy chain 4 (ITIH4) gene. J Hum Genet 2004;49:24–8. https://doi.org/10.1007/s10038-003-0101-8.

[18] Yao J, Jia L, Khan N, Lin C, Mitter SK, Boulton ME, Dunaief JL, Klionsky DJ, Guan JL, Thompson DA, et al. Deletion of autophagy inducer RB1CC1 results in degeneration of the retinal pigment epithelium. Autophagy 2015;11:939–53. https://doi.org/10.1080/15548627.2015.1041699.

[19] Goo YH, Son SH, Yechoor VK, Paul A. Transcriptional Profiling of Foam Cells Reveals Induction of Guanylate-Binding Proteins Following Western Diet Acceleration of Atherosclerosis in the Absence of Global Changes in Inflammation. Journal of the American Heart Association: Cardiovascular and Cerebrovascular Disease 2016;5. https://doi.org/10.1161/JAHA.115.002663.

[20] Shayo SC, Ogiso K, Kawade S, Hashiguchi H, Deguchi T, Nishio Y. Dietary obesity and glycemic excursions cause a parallel increase in STEAP4 and pro-inflammatory gene expression in murine PBMCs. Diabetol Int 2022;13:358–71. https://doi.org/10.1007/s13340-021-00542-1.

[21] Li Z, Liu J, Liu Z, Zhu X, Geng R, Ding R, Xu H, Huang S. Comprehensive analysis identifies crucial genes associated with immune cells mediating progression of carotid atherosclerotic plaque. Aging 2024;16:3880–95. https://doi.org/10.18632/AGING.205566.

[22] Zheng Y, Qi B, Gao W, Qi Z, Liu Y, Wang Y, Feng J, Cheng X, Luo Z, Li T. Macrophages-Related Genes Biomarkers in the Deterioration of Atherosclerosis. Front Cardiovasc Med 2022;9. https://doi.org/10.3389/FCVM.2022.890321.

[23] Liu R, Cao H, Zhang S, Cai M, Zou T, Wang G, Zhang D, Wang X, Xu J, Deng S, et al. ZBP1-mediated apoptosis and inflammation exacerbate steatotic liver ischemia/reperfusion injury. J Clin Invest 2024;134. https://doi.org/10.1172/JCI180451.

[24] Han J, Opoku E, Smith JD. Abstract 252: Is Zbp1 An Atherosclerosis Modifier Gene? Arterioscler Thromb Vasc Biol 2023;43. https://doi.org/10.1161/ATVB.43.SUPPL_1.252.

[25] Lin T-Y, Wei T-YW, Li S, Wang S-C, He M, Martin M, Zhang J, Shentu T-P, Xiao H, Kang J, et al. TIFA as a crucial mediator for NLRP3 inflammasome. Proc Natl Acad Sci U S A 2016;113:15078–83. https://doi.org/10.1073/pnas.1618773114.

[26] Demchev V, Malana G, Vangala D, Stoll J, Desai A, Kang HW, Li Y, Nayeb-Hashemi H, Niepel M, Cohen DE, et al. Targeted Deletion of Fibrinogen Like Protein 1 Reveals a Novel Role in Energy Substrate Utilization. PLoS One 2013;8:e58084. https://doi.org/10.1371/JOURNAL.PONE.0058084.

[27] Bazan HA, Brooks AJ, Vongbunyong K, Tee C, Douglas HF, Klingenberg NC, Woods TC. A pro-inflammatory and fibrous cap thinning transcriptome profile accompanies carotid plaque rupture leading to stroke. Sci Rep 2022;12:13499. https://doi.org/10.1038/S41598-022-17546-9.

[28] Ito S, Naito M, Kobayashi Y, Takatsuka H, Jiang S, Usuda H, Umezu H, Hasegawa G, Arakawa M, Shultz LD, et al. Roles of a macrophage receptor with collagenous structure (MARCO) in host defense and heterogeneity of splenic marginal zone macrophages. Arch Histol Cytol 1999;62:83–95. https://doi.org/10.1679/aohc.62.83.

[29] Amersfoort J, Schaftenaar FH, Douna H, van Santbrink PJ, Kröner MJ, van Puijvelde GHM, Quax PHA, Kuiper J, Bot I. Lipocalin-2 contributes to experimental atherosclerosis in a stage-dependent manner. Atherosclerosis 2018;275:214–24. https://doi.org/10.1016/J.ATHEROSCLEROSIS.2018.06.015.

[30] Shibata K, Sato K, Shirai R, Seki T, Okano T, Yamashita T, Koide A, Mitsuboshi M, Mori Y, Hirano T, et al. Lipocalin-2 exerts pro-atherosclerotic effects as evidenced by in vitro and in vivo experiments. Heart Vessels 2020;35:1012–24. https://doi.org/10.1007/S00380-020-01556-6/FIGURES/7.

[31] Hu S, Zhu Y, Zhao X, Li R, Shao G, Gong D, Hu C, Liu H, Xu K, Liu C, et al. Hepatocytic lipocalin-2 controls HDL metabolism and atherosclerosis via Nedd4-1-SR-BI axis in mice. Dev Cell 2023;58:2326-2337.e5. https://doi.org/10.1016/J.DEVCEL.2023.09.007.

[32] Riederer M, Erwa W, Zimmermann R, Frank S, Zechner R. Adipose tissue as a source of nicotinamide N-methyltransferase and homocysteine. Atherosclerosis 2009;204:412–7. https://doi.org/10.1016/J.ATHEROSCLEROSIS.2008.09.015.

[33] Hong S, Moreno-Navarrete JM, Wei X, Kikukawa Y, Tzameli I, Prasad D, Lee Y, Asara JM, Fernandez-Real JM, Maratos-Flier E, et al. Nicotinamide N-methyltransferase regulates hepatic nutrient metabolism through Sirt1 protein stabilization. Nat Med 2015;21:887. https://doi.org/10.1038/NM.3882.

[34] Webb NR, De Beer MC, Wroblewski JM, Ji A, Bailey W, Shridas P, Charnigo RJ, Noffsinger VP, Witta J, Howatt DA, et al. Deficiency of Endogenous Acute-Phase Serum Amyloid A Protects apoE-/- Mice From Angiotensin II-Induced Abdominal Aortic Aneurysm Formation. Arterioscler Thromb Vasc Biol 2015;35:1156–65. https://doi.org/10.1161/ATVBAHA.114.304776.

[35] Dong Z, Wu T, Qin W, An C, Wang Z, Zhang M, Zhang Y, Zhang C, An F. Serum amyloid A directly accelerates the progression of atherosclerosis in apolipoprotein E-deficient mice. Mol Med 2011;17:1357–64. https://doi.org/10.2119/MOLMED.2011.00186.

[36] Thompson JC, Jayne C, Thompson J, Wilson PG, Yoder MH, Webb N, Tannock LR. A brief elevation of serum amyloid A is sufficient to increase atherosclerosis. J Lipid Res 2015;56:286–93. https://doi.org/10.1194/JLR.M054015.
